# Prevalence and Subtypes Distribution (ST10, ST14, ST25, ST26) of *Blastocystis* spp. in Anatolian Water Buffalo (*Bubalus bubalis*) in Van, Türkiye

**DOI:** 10.1002/vms3.70054

**Published:** 2024-09-27

**Authors:** Adnan Ayan, Burcak Aslan Celik, Ozgur Yasar Celik, Ali Bilgin Yilmaz, Ozlem Orunc Kilinc, Ozge Oktay Ayan

**Affiliations:** ^1^ Department of Genetics Faculty of Veterinary Medicine Van Yuzuncu Yil University Van Turkey; ^2^ Department of Parasitology Faculty of Veterinary Medicine Siirt University Siirt Turkey; ^3^ Department of Internal Medicine Faculty of Veterinary Medicine Siirt University Siirt Turkey; ^4^ School of Health Van Yuzuncu Yil University Van Turkey; ^5^ Özalp Vocational School Van Yuzuncu Yil University Van Turkey; ^6^ Department of Parasitology Faculty of Medicine Van Yuzuncu Yil University Van Turkey

**Keywords:** Anatolian buffalo, blastocystis subtypes, Türkiye, Van

## Abstract

**Background:**

*Blastocystis* spp. is one of the most common protozoa worldwide, living in the gastrointestinal tract of humans and many other animals. On the basis of the genetic heterogeneity of small subunit ribosomal RNA, at least 28 subtypes (ST1‐ST17, ST21 and ST23–ST32) are reported to exist in mammals and birds.

**Objectives:**

This study was carried out to determine the prevalence and subtypes of *Blastocystis* spp. in Anatolian buffaloes (*Bubalus bubalis*) in Van province in the Eastern Anatolia Region of Turkey.

**Methods:**

DNA was extracted using commercial GeneMATRIX Stool DNA Purification Kit and then stored at −20°C until PCR amplification. After PCR amplification of the SSU rRNA gene region positive *Blastocystis* spp., amplicons from buffalo faeces were sequenced and then deposited in GenBank (OR576949.1, OR576950.1, OR576970.1, OR576971.1, OR577019.1, PP837943.1, PP837940.1, PP837939.1, PP837604.1, PP837937.1, PP837934.1, PP837601.1, PP837936.1 and PP837603.1).

**Results:**

PCR analysis of 120 faecal samples showed a total prevalence of 11.67% (14/120). The prevalence was higher in females and young animals (*p* > 0.05). Sequence analysis revealed *Blastocystis* spp., ST10, ST14, ST25 and ST26 subtypes. To our knowledge, *Blastocystis* subtypes ST25 and ST26 in buffaloes were reported for the first time in this study.

**Conclusion:**

It is thought that more large‐scale studies should be carried out to determine the zoonotic subtype potential of this protozoan in the region.

## Graphical abstract

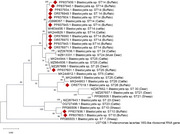

## 1 Introduction

*Blastocystis* spp. is one of the most common microorganisms infecting the gastrointestinal tract of a wide range of animals and humans (Duda, Stenzel, and Boreham 1998; Popruk, Pintong, and Radomyos 2013; Sreekumar et al. 2014; Zhu et al. 2017; Aslan Çelik 2023). Different forms of *Blastocystis* spp., including vacuolar, granular, amoeboid and cysts forms, have been reported (Popruk, Pintong, and Radomyos 2013; Hemalatha et al. 2014; Kamaruddin, Mat Yusof, and Mohammad 2020). Among these forms, the faecal cyst is the only environmentally resistant transmissible form (Hemalatha et al. 2014). Transmission of the agent is via the faecal–oral route through contaminated water and food (Noradilah et al. 2017; Moura et al. 2018; Tavur and Önder 2022; Aslan Çelik 2023).

It is reported that pathogenicity in this disease varies depending on the subtype of the parasite and the immune status of the individuals (Aynur et al. 2019). Some studies conducted in different parts of the world indicate that the disease has zoonotic potential (Tan 2008; AbuOdeh et al. 2019). Close contact between humans and animals may pose a risk for zoonotic transmission of *Blastocystis* (Onder, Yildirim, and Pekmezci 2021). On the basis of the genetic heterogeneity of small subunit ribosomal RNA (SSU rRNA), at least 28 subtypes (ST1‐ST17, ST21 and ST23–ST32) are reported to exist in mammals and birds (Jinatham et al. 2021; Ren et al. 2023). So far, ST1‐ST9 and ST12 (Jiménez, Jaimes, and Ramírez 2019; Jinatham et al. 2021), as well as a single instance of ST10, ST14 and ST16, have been reported in humans (Jinatham et al. 2021).

In the worldwide, studies on the prevalence of *Blastocysts* spp. in buffaloes are quite limited, with 62% prevalence reported in China (Li et al. 2022), 21.05% in Nepal (Lee et al. 2012), 17.82% in Italy (Gabrielli et al. 2021) and 17%–35% in Turkey (Onder, Yildirim, and Pekmezci 2021; Çelik et al. 2022). This study was carried out to determine the prevalence and subtypes distribution of *Blastocystis* spp. in Anatolian buffaloes in Van province, Türkiye.

## 2 Materials and Methods

### 2.1 The Study Area and Sample Collection

This study was conducted in Van province located in the Eastern Anatolia region of Türkiye (38°31′54″ N, 43°24′47″ E) between September 2023 and April 2024. The animal material of the study consisted of 120 randomly selected clinically healthy Anatolian buffaloes in different farms. The faecal samples taken from the rectum of the animals were placed in individual sample containers, and age and sex information of each buffalo were recorded.

### 2.2 DNA Extraction

DNA was extracted using commercial GeneMATRIX Stool DNA Purification Kit (EURx, Poland) according to the manufacturer's instruction and then stored at −20°C until PCR amplification.

### 2.3 PCR Amplification

For amplification of the SSU rRNA gene region of *Blastocystis* spp., forward blast (5′‐GGA GGT AGT GAC AAT AAA TC‐3′) and reverse blast (5′‐TGC TTT CGC ACT TGT TCA TC‐3′) primers previously reported by Santín et al. (2011) were used. Each 20‐µL PCR mixture contained 4 µL of 5× FIREPol Master Mix (7.5 mM MgCl_2_, Solis BioDyne, Estonia), 8 pmol of each primer, 1.6 µL of DNA and 12.8 µL nuclease‐free water. The reaction was followed by pre‐denaturation at 95°C for 5 min, each cycle consisted of 35 cycles of denaturation (95°C for 30 s), annealing (54°C for 30 s) and elongation (72°C for 1 min) with a final elongation of 5 min at 72°C. PCR products were analysed on 1.5% agarose gel, visualized by Safe‐T‐Stain (BioShop, Canada).

### 2.4 Sequence Analysis and Phylogeny

Positive *Blastocystis* spp. amplicons from buffalo faeces (*n* = 14) were sequenced on an Applied Biosystems 3500 Genetic Analyzer (Thermo Fisher Scientific, MA, USA) by BM Labosis Company (Ankara/Turkey). Sequencing results were first analysed using the sequencing programme Bioedit (Hall 1999) and then deposited in GenBank (NCBI) as OR576949.1, OR576950.1, OR576970.1, OR576971.1, OR577019.1, PP837943.1, PP837940.1, PP837939.1, PP837604.1, PP837937.1, PP837934.1, PP837601.1, PP837936.1 and PP837603.1.

The phylogenetic relationship between species was made using the T92 and G models determined by J Model Test 2.0 for SSU rRNA alignments. Alignments of the obtained SSU rRNA sequence with closely related sequences in the GenBank database and phylogenetic tree structure were performed using the Mega 11 programme (Tamura, Stecher, and Kumar 2021) based on the Maximum Likelihood Method. *Proteromonas lacertae* (U37108.1) was used as outgroup.

### 2.5 Statistical Analysis

The data obtained in the study were analysed using the SPSS V16.0 (IBM, Chicago, IL, USA) programme. The relationship between grouped variables was calculated using chi‐square test. The difference was considered statistically significant when *p* < 0.05.

## 3 Results

**FIGURE 1:**
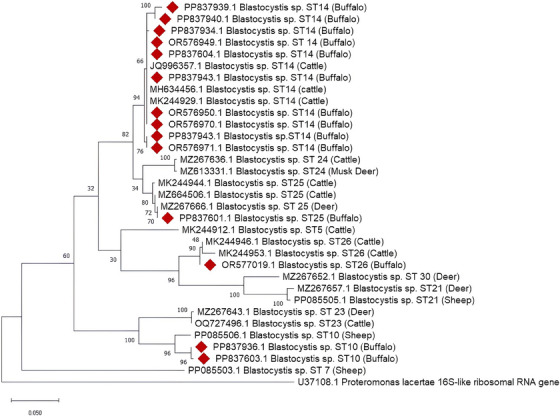
Phylogenetic relationships of *Blastocystis* sp. isolates from buffalo faeces using maximum likelihood method and analysis based on small subunit ribosomal RNA (SSU rRNA) gene region. Bootstrap value of 1000 replicates was selected. *Proteromonas lacertae* (U37108.1) was used as outgroup.

As a result of PCR analysis, the total prevalence in buffaloes was determined as 11.67% (14/120) (Table 1). In this study, higher prevalence was found in females than males. According to age, higher prevalence was found in those younger than one year (*p* > 0.05). According to the sequence results, 2 samples were identified as *Blastocystis* ST10 (PP837936.1 and PP837603.1), 10 samples were identified as ST14 (OR576949.1, OR576950.1, OR576971.1, OR576970.1, PP837943.1, PP837940.1, PP837939.1, PP837604.1, PP837937.1 and PP837934.1), 1 sample was identified as ST25 (PP837601.1), and 1 sample was identified as ST26 (OR577019.1). The sequences obtained were deposited in GenBank. BLAST analysis showed that the *Blastocystis* species obtained in this study had high similarity compared to the data sets in GenBank (Table 2). Phylogenetic analysis of SSU rRNA gene sequences confirmed *Blastocystis* spp. STs in this study (Figure 1).

**TABLE 1:** Prevalence and distribution of *Blastocystis* spp. according to age and gender.

Categories	(*n*)	Positive		*p*
		(*n*)	(%)	
**Age**				
0–6	44	7	15.91	0.35
7–12	36	5	13.89	
13–18	24	2	8.33	
19–48	16	0	0.00	
**Gender**				
Female	84	11	13.10	0.46
Male	36	3	8.33	
**Overall**	120	14	11.67	

**TABLE 2:** The DNA sequences deposited in GenBank as a result of this study.

Obtained sequences			Reference sequences from GenBank			
Pathogen	Accession number	Length (bp)	Locality	Host	Identity (%)	Accession number
*Blastocystis* sp. ST10	PP837936.1	434	Turkey China	Calf Sheep	99.54 91.99	OM920832.1 PP447149.1
*Blastocystis* sp. ST10	PP837603.1	405	China USA	Sheep Cattle	91.96 91.87	PP447149.1 MK244920.1
*Blastocystis* sp. ST14	OR576949.1	484	USA USA	Cattle Cattle	100 100	MH634456 JQ996357
*Blastocystis* sp. ST14	OR576950.1	484	USA Spain	Cattle Cattle	100 100	MK244929 MZ664508
*Blastocystis* sp. ST14	OR576971.1	484	USA USA	Cattle Cattle	100 100	JQ996357 MH634456
*Blastocystis* sp. ST14	OR576970.1	461	Spain USA	Cattle Cattle	100 100	MZ664508 MK244929
*Blastocystis* sp. ST14	PP837943.1	480	Spain USA	Cattle Cattle	99.79 99.79	MZ664521 MK244931
*Blastocystis* sp. ST14	PP837940.1	436	Spain USA	Cattle Cattle	100 100	MZ664508 MK244929
*Blastocystis* sp. ST14	PP837939.1	443	Spain USA	Cattle Cattle	100 100	MZ664508 MK244929
*Blastocystis* sp. ST14	PP837604.1	430	England USA	Cattle Cattle	100 100	KC148205.1 OR117668.1
*Blastocystis* sp. ST14	PP837937.1	446	South Korea USA	Dog Cattle	99.29 99.10	ON908959.1 MH634456
*Blastocystis* sp. ST14	PP837934.1	442	USA Spain	Cattle Cattle	99.09 99.09	MK244929 MZ664508
*Blastocystis* sp. ST25	PP837601.1	449	China Belgium	Yak Sheep	99.55 99.53	PP396173.1 HF569213
*Blastocystis* sp. ST26	OR577019.1	408	China USA	Cattle Cattle	99.64 99.18	MW737697.1 MK244946.1

## 4 Discussion

*Blastocystis* spp. is a common protist living in the gastrointestinal tract of human and other animal hosts (Jinatham et al. 2021). Despite more than 100 years since *Blastocystis* was first described and the numerous of research studies, there are still many unknowns about this organism (Stensvold and Clark 2016).

Microscopic, culture and molecular methods are used in the diagnosis of *Blastocystis* and other parasitic agents (Ertuğ et al. 2015; Koca, Çelik, et al. 2023; Koca, Kılınç, et al. 2023). Among these, molecular method is reported to be more sensitive and specific (Stensvold, Alfellani, and Nørskov‐Lauritsen 2009; Malatyali and Özçelik 2011). Therefore, PCR method was used in this study. As a result of this study, a prevalence of 11.67% (14/120) was determined. The results of this study were lower than the results of the studies conducted by Li et al. (2022) in China (62%), Lee et al. (2012) in Nepal (21.05%) and Onder, Yildirim, and Pekmezci (2021) in Turkey (35%), and concur the results of the studies conducted by Gabrielli et al. (2021) in Italy (17.82%) and Çelik et al. (2022) in Turkey (17%). The reasons for the differences between the studies can include geographical location, sampling season, number of animals, age of animals, immune status of animals, care and feeding conditions, stress and methods used (Zhu et al. 2017; Lee et al. 2018; Onder, Yildirim, and Pekmezci 2021).

It is reported that ST3, ST4, ST5, ST10, ST10, ST12, ST13, ST13, ST14, ST17, ST21–ST26 and ST32 subtypes have been identified in studies in which *Blastocystis* subtypes were determined in cattle in the world (Aynur et al. 2019; Maloney et al. 2019; Higuera, Herrera, and Jimenez 2021; Onder, Yildirim, and Pekmezci 2021; Öner et al. 2022). Studies in which *Blastocystis* subtypes were determined in buffaloes are quite limited. It was reported that ST10 was detected by Li et al. (2022) in China, ST14 was detected by Gabrielli et al. (2021) in Italy and Onder, Yildirim, and Pekmezci (2021) in Turkey and ST4 by Lee et al. (2012) in Nepal. Although ST1–ST9 and ST12 subtypes of *Blastocystis* spp. have been reported to occur in humans (Stensvold, Alfellani, and Nørskov‐Lauritsen 2009; Zhu et al. 2017; AbuOdeh et al. 2019), ST10, ST14 and ST16 have also been reported (Jinatham et al. 2021). Studies conducted in Turkey reported that blastocystis ST1–ST7 was detected (Büyükbaba et al. 2017; Malatyali, Ertabaklar, and Ertuğ 2023). As a result of this study, *Blastocystis* subtypes ST10 (14.29%), ST14 (71.43%), ST25 (7.14%) and ST26 (7.14%) were identified from buffaloes. To our knowledge, *Blastocystis* subtypes ST25 and ST26 in buffaloes were reported for the first time by this study. Cattle populations are widespread in the areas where these samples were taken. The reason why these subtypes (ST10, ST25 and ST26) are seen in buffaloes may be due to their close relationship with cattle. In this study, ST10 sequences obtained as a result of BLAST analysis showed 99.54%, 91.99% and 91.87% similarity with sequences obtained from calf in Turkey (OM920832.1), sheep in China (PP447149.1) and cattle in the USA (MK244920.1), respectively. The ST14 sequence revealed 100% similarity with sequences obtained from cattle in the USA (MH634456, MK244929, JQ996357 and JQ996357) and Spain (MZ664508). The ST25 sequence was 99.55% and 99.53% overlap with sequences obtained from yak in China (PP396173.1) and sheep in Belgium (HF569213) respectively. The ST26 sequence showed 99.64% and 99.18% similarity with sequences obtained from cattle in China (MW737697.1) and the USA (MK244946.1). Among the subtypes obtained in this study, ST10 and ST14 subtypes, which have zoonotic properties, were not detected in humans in Turkey, indicating that there is no source of transmission from buffaloes to humans. However, studies involving more populations in humans should be conducted.

Previous studies conducted in Australia (Duda, Stenzel, and Boreham 1998), Malaysia (Kamaruddin, Mat Yusof, and Mohammad 2020) and Turkey (Çelik et al. 2022) reported higher prevalence in females. In this study, similar to other researchers, higher prevalence was found in females compared to males (*p* > 0.05). Whether gender is a contributing factor to the distribution of infection has not yet been clarified. More research is reportedly needed for this (Kamaruddin, Mat Yusof, and Mohammad 2020).

Previous studies in Turkey (Tavur and Önder 2022) and Iran (Daryani et al. 2008) reported a higher prevalence in adults, whereas studies in Malaysia (Kamaruddin, Mat Yusof, and Mohammad 2020) and Korea (Lee et al. 2018) found a higher prevalence in young individuals. In this study, a higher prevalence was found in young individuals, and this result supports the findings of other researchers (Lee et al. 2018; Kamaruddin, Mat Yusof, and Mohammad 2020) (*p* > 0.05).

In conclusion, this study revealed the prevalence of *Blastocystis* spp. as well as the presence of ST10, ST14, ST25 and ST26 in buffaloes in Van province. As ST10 and ST14 have been reported in humans, this suggests that it may be a source of infection for human *Blastocystis* through direct contact with buffaloes or contamination of the water source. Large‐scale studies are recommended to determine the zoonotic subtype potential of the agent in the region.

## Author Contributions

**Adnan Ayan**: conceptualization, methodology, formal analysis, funding, investigation, writing–original draft preparation, writing‐review and editing. **Burcak Aslan Celik**: conceptualization, funding, formal analysis, investigation, writing–original draft preparation, writing–review and editing. **Ozgur Yasar Celik**: methodology, funding, investigation, writing–original draft preparation, writing–review and editing. **Ali Bilgin Yilmaz**: methodology, writing–review and editing. **Ozlem Orunc Kilinc**: formal analysis, investigation. **Ozge Oktay Ayan**: conceptualization, writing‐original draft preparation, writing‐review and editing, formal analysis, investigation.

## Ethics Statement

This study was approved by Van Yuzuncu Yil University Animal Experiments Local Ethics Committee (Approval No: 2023/10‐11).

## Conflicts of Interest

The authors declare no conflicts of interest.

## Data Availability

The data sets generated for this study are available on request to the corresponding author.
